# Type IV collagen reduces mucin 5AC secretion in three-dimensional cultured human primary airway epithelial cells

**DOI:** 10.1016/j.bbrep.2019.100707

**Published:** 2019-11-08

**Authors:** Yuho Ito, Jun Iwashita, Jun Murata

**Affiliations:** a Environmental Protection Division, Research Center for Public Health and Environment, Akita, Japan; b Faculty of Bioresource Sciences, Akita Prefectural University, 241-438 Kaidobata-Nishi, Shimoshino-Nakano, Akita, Akita, 010-0195, Japan

**Keywords:** Asthma, Extracellular matrix, Type IV collagen, MUC5AC, ERK

## Abstract

Mucin 5AC (MUC5AC) hypersecretion induces airway narrowing in patients with asthma, which leads to breathing problems. We investigated the regulation of MUC5AC secretion by extracellular matrix (ECM) proteins in human primary airway epithelial cells from patients with asthma. The addition of type IV collagen to three-dimensional cultured human primary airway epithelial cells, which mimics the airway surface, reduced MUC5AC secretion in the medium, while the addition of laminin increased MUC5AC secretion. Furthermore, the addition of fibronectin did not affect MUC5AC secretion. In particular, the repeated addition of a low concentration of type IV collagen demonstrated a cumulative effect on the reduction in MUC5AC secretion. Human primary cells incubated with type IV collagen showed downregulated extracellular signal-regulated kinase (ERK) activity, which induced MUC5AC hypersecretion but did not affect Akt activity. These results suggest that the addition of type IV collagen to the apical surface of primary cells downregulates MUC5AC secretion and has a cumulative effect on MUC5AC secretion which might be effected via the ERK signaling pathway.

## Highlights

• We studied the regulation of MUC5AC secretion by extracellular matrix proteins.
• The addition of type IV collagen reduced MUC5AC secretion in primary airway cells.
• The repeated addition of a low concentration of collagen reduced MUC5AC secretion.
• Type IV collagen induced the downregulation of ERK which induces MUC5AC secretion.

## Graphical abstract

**Figure:**
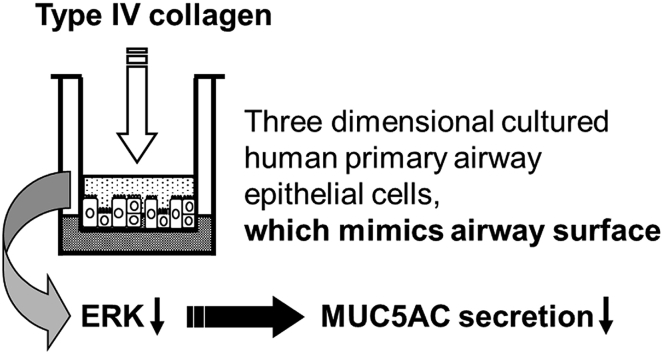

## 1 Introduction

The mucus layer in human airways acts as a protective barrier against foreign irritants and is an indispensable primary host defense. Asthma, also referred to as bronchial asthma, is a chronic lung disease caused by inflammation and narrowing of the bronchial tubes. Airway mucus hypersecretion, which induces airway narrowing and disease exacerbation, is a characteristic feature in patients with asthma [1,2]. Therefore, the regulation of airway mucus secretion is important for its treatment.

Mucins, which are large, highly glycosylated proteins with tandemly repeating sequences, are the major constituents of airway mucus and are secreted by goblet cells or submucosal glands in the airway epithelia [3]. Approximately 20 different mucin gene subfamilies have been identified to date. *MUC1*, *MUC2*, *MUC4*, *MUC5AC*, *MUC5B*, *MUC7*, *MUC8*, and *MUC13* are expressed at the messenger RNA level in human airways [4]. Mucin 5B and mucin 5AC (MUC5AC) are the primary gel-forming mucins in the mucus layer in normal human airways. MUC5AC hypersecretion owing to increased airway mucus is a characteristic feature in patients with asthma [[5], [6], [7], [8]].

MUC5AC secretion is regulated by parasympathetic nervous system stimulation [9]. Several in vitro and in vivo studies have described the regulation of *MUC5AC* expression in human primary airway epithelial cells as a potential therapeutic target in asthma [5]. Bacterial inflammation, cell–cell adhesion, protein kinase B, and certain flavonoids in human airways induce morphological and proliferative changes in goblet cells in the airway epithelia, which results in airway mucus hypersecretion [[10], [11], [12]]. Several proinflammatory cytokines, including interleukins (IL)-1β, IL-6, and IL-17, upregulate *MUC5AC* expression in human primary airway epithelial cells [13,14], and the majority of the signals that induce MUC5AC secretion are mediated by the activation of epidermal growth factor (EGF) receptors [15,16]. Further, EGF receptors activate the extracellular signal-regulated kinase (ERK) signaling pathway, which results in increased NF-κB and Sp1 transcription factors, followed by *MUC5AC* upregulation [17]. Akt, also known as protein kinase B, is a serine/threonine kinase that is phosphorylated and activated by the integrin pathway. It plays important roles in numerous cellular functions, such as cell proliferation, cell migration, and gene transcription [18,19]. In our previous report, it was shown that Akt induced the downregulation of MUC5AC production and Akt was activated by type IV collagen in the human epithelial cell line NCI–H292 [11].

In our previous study, certain ECM proteins were reported to be involved in the regulation of MUC5AC secretion. The ECM contains several proteins, such as laminins, fibronectins, and collagens, which provide structural support and regulation to the surrounding cells [[20], [21], [22], [23], [24]]. Laminins are involved in the in vivo formation of ECM structure in the basal laminae. Fibronectins are glycoproteins which play a significant role in cell migration. Collagens are the most abundant proteins in the ECM which provide structural support to resident cells, such as human airway epithelial cells. Type IV collagen is abundant in the basement membrane and plays a role in cell–cell communication. We previously reported that MUC5AC secretion was upregulated NCI–H292 cells when cultured in plates coated with laminin, while downregulated when cultured with type IV collagen [25]. However, the effect of ECM proteins on human primary airway epithelial cells in patients with asthma remains unclear, which resembles its effect on three-dimensional cultured human primary airway epithelial cells.

In this study, the regulation of MUC5AC secretion by ECM proteins in human primary airway epithelial cells was investigated. Our results suggest that type IV collagen downregulates MUC5AC secretion in three-dimensional cultured human primary airway epithelial cells derived from patients with asthma.

## 2 Experimental procedures

### 2.1 Cell culture

Human airway epithelia consisting of primary epithelial cells, MucilAir (EP03MD, Epithelix Sàrl, Geneva, Switzerland), is a three-dimensional model of differentiated human epithelium. The MucilAir primary cells were maintained according to the manufacturer's protocol. In brief, the airway primary cells derived from asthmatic patients were cultured at the air-liquid interface in 700 μL of culture medium in cell culture chambers. The cells were maintained in a 5% CO2 incubator at 37 °C at the air-liquid interface with fresh medium replaced every 3 days. Human airway cancer cell line NCI–H292 was purchased from the American Type Culture Collection (Manassas, VA, USA). NCI–H292 cells were cultured in RPMI-1640 (Sigma-Aldrich, Tokyo, Japan) supplemented with 10% fetal bovine serum (FBS, Cansera International, Etobicoke, Ontario, Canada), 100 units/mL of penicillin (Gibco Oriental, Tokyo, Japan), and 100 μg/mL streptomycin (Gibco Oriental) in a 5% CO2 incubator at 37 °C. Adherent cells were subcultured every 3–4 days by treatment with a trypsin–EDTA solution (Gibco Oriental).

### 2.2 Reagents

Resazurin (Funakoshi, Tokyo, Japan) was prepared as an aqueous stock solution (4 mM) in distilled water, sterilized by membrane filtration, and stored at −20 °C until required. U0126 (Wako, Tokyo, Japan), an inhibitor of the MEK/ERK pathway, was dissolved in 10 mM in dimethylsulfoxide (DMSO).

### 2.3 Cell proliferation assay

Mucilair, a human lung primary cells, chambers were incubated with 100 μL of 6 μM resazurin for 1 h at 37 °C, and the cell growth was assessed by measuring the absorbance at 570 nm with a microplate spectrophotometer Benchmark plus (BioRad). In NCI–H292 cells, cell proliferation was assessed by a Cell Counting Kit-8 (Dojindo, Kumamoto, Japan). NCI–H292 cells (1 × 10^4^ cells in 0.1 mL) were cultured on a 96-well plate (Sumilon, Tokyo, Japan) at 37 °C. The reagent of the kit (0.01 mL) was added to each well, and then the plate was incubated for 2 h at 37 °C. Cell growth was assessed by measuring the absorbance at 450 nm with a microplate spectrophotometer Benchmark plus (BioRad).

### 2.4 Incubation of cells with ECM proteins

The ECM proteins were dissolved in phosphate-buffered saline (PBS; 0.01 mM phosphate buffer, 0.138 mM NaCl, 0.0027 mM KCl, pH 7.4) and stored at −80 °C until required. The primary cells were cultured with indicated concentrations of 100 μL each of type IV collagen (Sigma, Tokyo, Japan), fibronectin (Asahi Techno Glass Corp., Tokyo, Japan), and laminin (BD Biosciences, CA, USA) for 6 h at 37 °C, and the NCI–H292 cells were cultured similarly for 30 h at 37 °C. The NCI–H292 cells that were cultured with ECM proteins (33 μg/mL) were incubated for 30 h at 37 °C, which were the conditions used in our previous study [25].

### 2.5 MUC5AC mucin protein assay

Mucilair primary cells, human primary cells, were added 100 μL of culture medium which contains ECM proteins for 6 h at 37 °C to its upper phase of overlapped cell layer and culture medium was sampled. NCI–H292 cells were washed once with culture medium and suspended in the medium by means of syringe with a 26G needle to create a single-cell suspension. Diluted cells (1 × 10^4^ cells per 100 μL) were added to the wells of 96-well plate and cultured in 100 μL of culture medium with ECM proteins for 30 h at 37 °C, and culture medium was sampled. A total of 2 μL of the sample was blotted onto an Immobilon membrane (Millipore, Bedford, MA, USA) by Dot Blot Hybridization Manifold (48 wells; SCIE-PLAS, Cambridge, UK). The membrane was treated with 4% skim milk (Gibco Oriental) in 0.1% Tween 20-TBS (TBS-T) for 12 h at 4 °C, and then incubated with mouse anti-human MUC5AC antibody (1:2000 in 4% skim milk, MS145-P1, Thermo Scientific, Kanagawa, Japan) for 1 h. The membrane was washed five times for 5 min each with TBS-T and then incubated with rabbit anti-mouse IgG (H + L) (1:2000 in 4% skim milk, NA931V, GE Healthcare, Buckinghamshire, UK) for 1 h. After being washed five times for 5 min each with TBS-T, enzyme reactions were detected with a Luminata Forte western HRP substrate (WBLUF0500, Millipore) and a Chemidoc image analyzer (Biorad, Tokyo, Japan).

### 2.6 Inhibition of MEK/ERK

U0126 was added to the cell culture medium to a final concentration of 10 μM and cultured for 6 h at 37 °C. The same concentration of DMSO was added to the controls.

### 2.7 Western blot analysis

The treated primary cells cultured in chambers were lysed in a conventional SDS sample buffer (62.5 mM Tris, 10% glycerol, 2% SDS, 0.01% bromophenol blue, pH 6.8). The samples were quantified with XL-Bradford kit (Aproscience, Tokushima, Japan). The 30 μg of samples were electrophoresed on 10% of acrylamide gels with a CM-1005 gel apparatus (Cima Biotech, Tokyo, Japan), and then blotted onto a Hybond ECL nitrocellulose membrane (GE Healthcare) with a Trans blot SD cell (Biorad). The membrane was treated with 4% skim milk (Gibco Oriental) in TBS-T (0.1% tween-20, 150 mM NaCl and 10 mM Tris pH 7.5) for 12 h at 4 °C and then incubated with a rabbit p-ERK 1/2 (Thr 202) (sc-101760, SANTA CRUZ BIOTECHNOLOGY, CA, USA), a mouse total ERK antibody (Sigma, Tokyo, Japan), a rabbit anti-phosphoAkt (Ser 473) antibody (4058, Cell Signaling Technology, Tokyo) or a mouse anti-total Akt (pan) antibody (2920S, Cell Signaling Technology) at a dilution of 1:2000 in 4% skim milk for 12 h at 4 °C. The membrane was washed five times for 5 min each with TBS-T and then incubated with an anti-rabbit IgG conjugated with horseradish peroxidase (W4018, Promega, Madison, WI, USA) or an anti-mouse IgG conjugated with horseradish peroxidase (W4028, Promega) at a dilution of 1:2000 in 4% skim milk for 1 h. After washing the membrane for five times for 5 min each with TBS-T, the enzyme reaction was detected with a Luminata Forte western HRP substrate (WBLUF0500, Millipore) and a Chemidoc image analyzer (Biorad, Tokyo, Japan). After detection, a blot membrane was incubated with Restore Western blot Stripping buffer (21059, Thermo Scientific, Rockford, IL, USA) for 15 min at room temperature with shaking. The membrane was washed five times for 5 min each with TBS-T and then treated with 4% skim milk (Gibco Oriental) in TBS-T for 12 h at 4 °C for reblocking.

### 2.8 Statistics

Analysis of variance (ANOVA) was used for comparisons among more than two groups. For other statistics, Student's t-test was performed. *p < 0.05 was considered significant.

## 3 Results

### 3.1 The effects of type IV collagen on MUC5AC secretion in primary cells

**Fig. 1:**
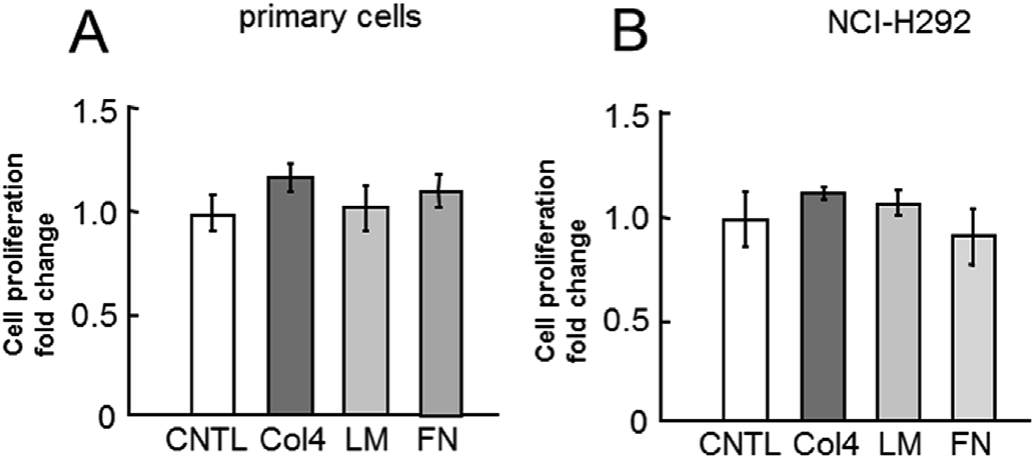
Evaluation of cell viability of human primary airway cells and NCI–H292 cell line cultured with type IV collagen, laminin, and fibronectin. Cells were incubated with PBS (CNTL), 500 μg/mL of type IV collagen (Col4), laminin (LM), or fibronectin (FN). The cell viability was analyzed using the cell proliferation assay. A, Human airway primary cells were cultured with ECM proteins for 6 h. Fold changes were based on CNTL level (mean ± SD, n = 6, one-way ANOVA). The representative results of 3 independent experiments are shown. B, NCI–H292 cells (1 × 10^4^ cells/well) were cultured with ECM proteins for 30 h. Fold changes were based on CNTL level (mean ± SD, n = 5, one-way ANOVA). The representative results of 3 independent experiments are shown.

The three-dimensional cultured primary airway epithelial cells from patients with asthma showed constitutive MUC5AC secretion into the culture medium. The incubation of cells with type IV collagen, laminin, and fibronectin for 6 h has no considerable effect on their viability (Fig. 1).

**Fig. 2:**
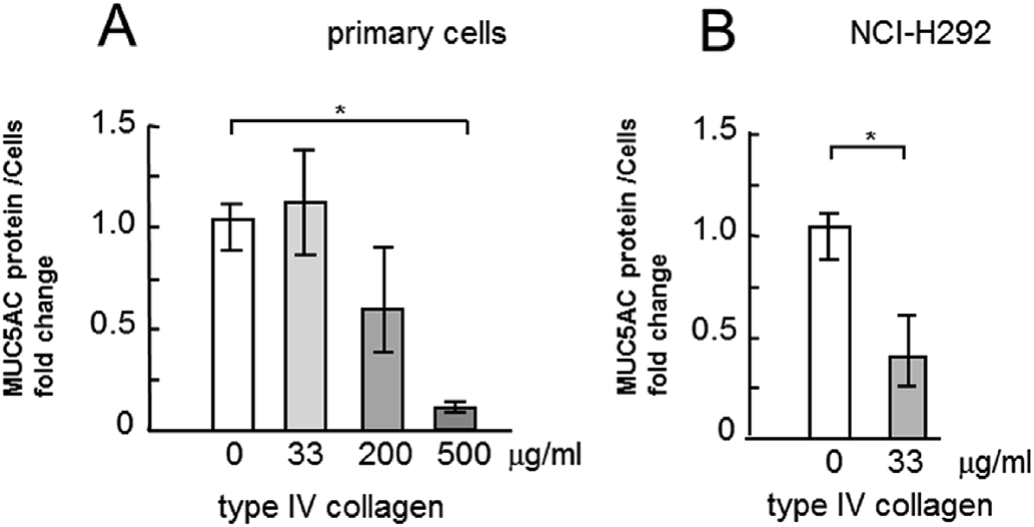
Evaluation of MUC5AC secretion in human airway primary cells and NCI–H292 cell line cultured with type IV collagen. A, Human airway primary cells were cultured in chambers (six wells) with type IV collagen (0, 33, 200, 500 μg/mL) for 6 h and then the culture medium were collected and sampled. The samples were analyzed using the mucin protein assay to detect the levels of MUC5AC protein. Fold changes were based on control level of secreted MUC5AC (0 μg/mL of type IV collagen) in medium (mean ± SD, n = 6, one-way ANOVA). The representative results of 3 independent experiments are shown. B, NCI–H292 cells (1 × 10^4^ cells/well) were cultured with 33 μg/mL of type IV collagen for 30 h in 96-well plates and then the culture medium were sampled. The samples were analyzed using the mucin protein assay to detect the levels of MUC5AC protein. Fold changes were based on control level (0 μg/mL of type IV collagen) of secreted MUC5AC in medium (mean ± SD, n = 5, one-way ANOVA). The representative results of 3 independent experiments are shown.

**Fig. 3:**
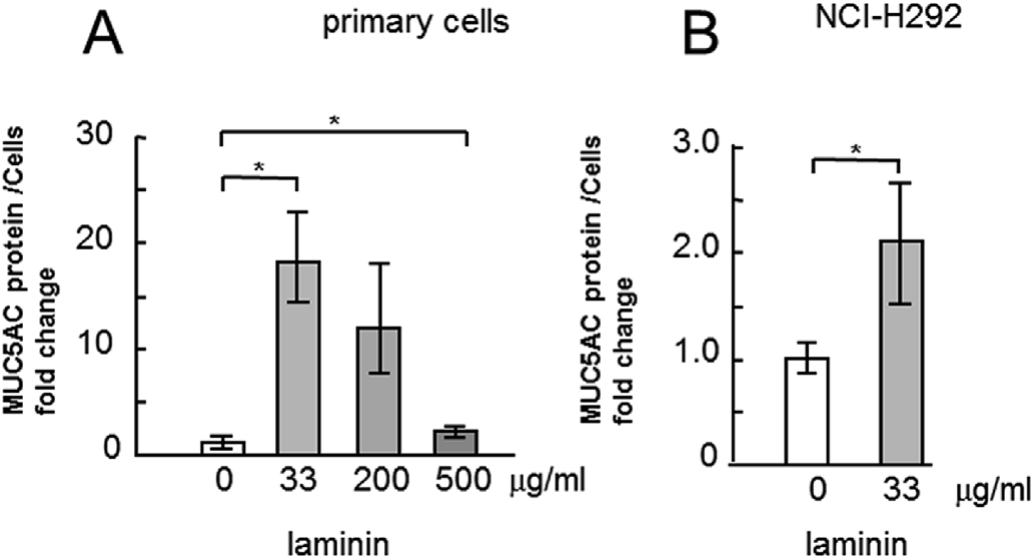
Evaluation of MUC5AC secretion in human airway primary cells and NCI–H292 cell line cultured with laminin. A, Human airway primary cells were cultured in chambers (six wells) with laminin (0, 33, 200, 500 μg/mL) for 6 h and then the culture medium were collected and sampled. The samples were analyzed using the mucin protein assay to detect the levels of MUC5AC protein. Fold changes were based on control level of MUC5AC (0 μg/mL of laminin) in cells (mean ± SD, n = 6, one-way ANOVA). The representative results of 3 independent experiments are shown. B, NCI–H292 cells (1 × 10^4^ cells/well) were cultured with 33 μg/mL of laminin for 30 h in 96-well plates and then the culture medium were sampled. The samples were analyzed using the mucin protein assay to detect the levels of MUC5AC protein. Fold changes were based on control level of secreted MUC5AC (0 μg/mL of laminin) in medium (mean ± SD, n = 5, one-way ANOVA). The representative results of 3 independent experiments are shown.

**Fig. 4:**
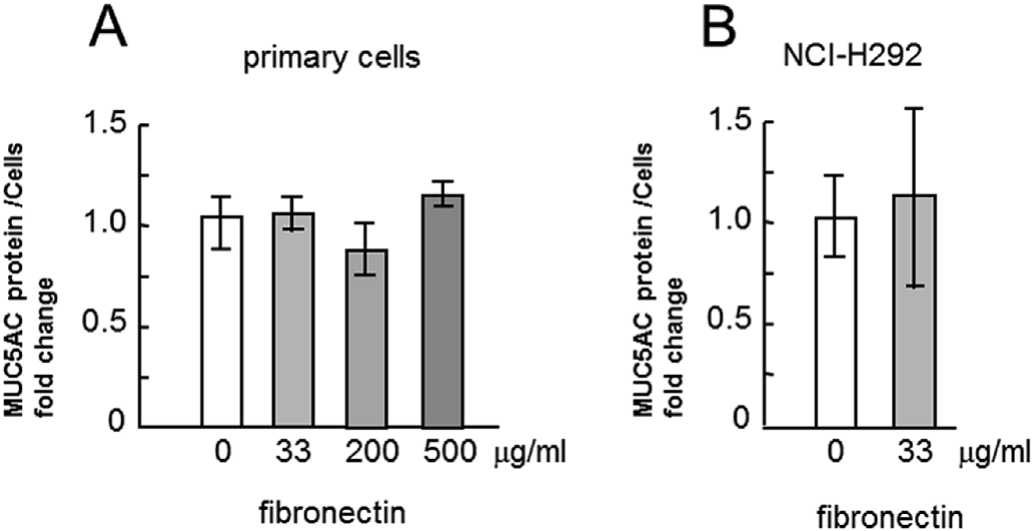
Evaluation of MUC5AC secretion in human airway primary cells and NCI–H292 cell line cultured with fibronectin. A, Human airway primary cells were cultured in chambers (six wells) with fibronectin (0, 33, 200, 500 μg/mL) for 6 h and then the culture medium were collected and sampled. The samples were analyzed using the mucin protein assay to detect the levels of MUC5AC protein. Fold changes were based on control level of secreted MUC5AC (0 μg/mL of fibronectin) in medium (mean ± SD, n = 6, one-way ANOVA). The representative results of 3 independent experiments are shown. B, NCI–H292 cells (1 × 10^4^ cells/well) were cultured with 33 μg/mL of fibronectin for 30 h in 96-well plates and then the culture medium were sampled. The samples were analyzed using the mucin protein assay to detect the levels of MUC5AC protein. Fold changes were based on control level of secreted MUC5AC (0 μg/mL of fibronectin) in medium (mean ± SD, n = 5, one-way ANOVA). The representative results of 3 independent experiments are shown.

The incubation of cells with type IV collagen induced a statistically significant and dose-dependent reduction in MUC5AC secretion into the culture medium (Fig. 2A), while the incubation of cells with laminin induced a considerable dose-dependent increase in MUC5AC secretion (Fig. 3A). However, the incubation of cells with fibronectin did not induce considerable changes in MUC5AC secretion (Fig. 4A). The MUC5AC secretion from cells incubated with type IV collagen, laminin, and fibronectin showed the same tendency in the NCI–H292 cells (Fig. 2, Fig. 3, Fig. 4B).

### 3.2 The effects of repeated addition of low levels of type IV collagen on MUC5AC secretion in primary cells

**Fig. 5:**
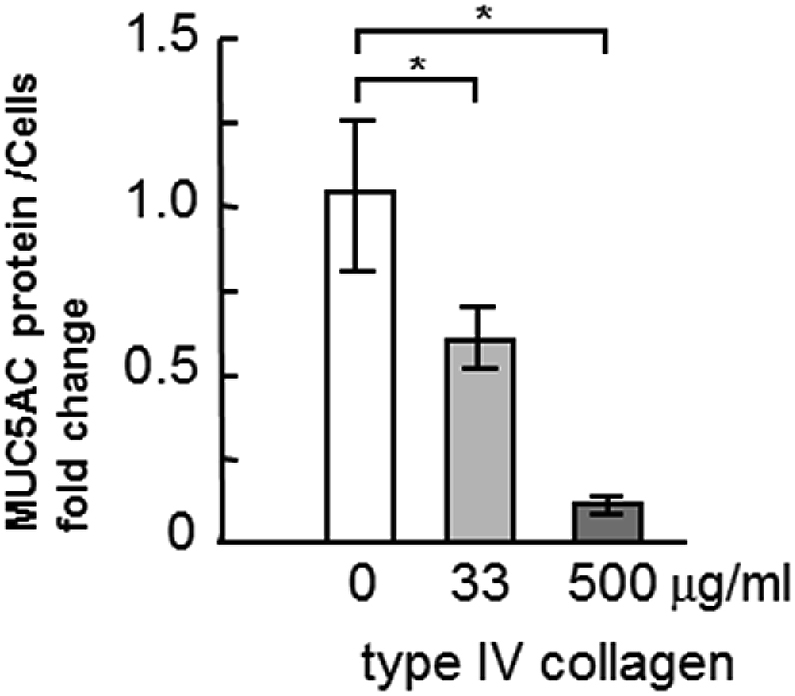
Evaluation of MUC5AC secretion in human airway primary cells which repeatedly cultured in the presence of type IV collagen. Human airway primary cells were cultured in chambers (six wells) with type IV collagen (0, 33, 500 μg/mL) for 6 h per day over a period of two weeks and then culture medium were collected and sampled. The samples were analyzed using the mucin protein assay to detect the levels of MUC5AC protein. Fold changes were based on control level (0 μg/mL of type IV collagen) of secreted MUC5AC in medium (mean ± SD, n = 6, one-way ANOVA). The representative results of 3 independent experiments are shown.

Reduction in MUC5AC secretion induced by type IV collagen is important in treating asthma. In this study, the cells were repeatedly incubated with a low concentration (33 μg/mL) of type IV collagen, for 6 h per day for 2 weeks, which was insufficient to reduce MUC5AC secretion. This repeated incubation induced a significant reduction in MUC5AC secretion (Fig. 5).

### 3.3 ERK and Akt activity in primary cells incubated with type IV collagen

**Fig. 6:**
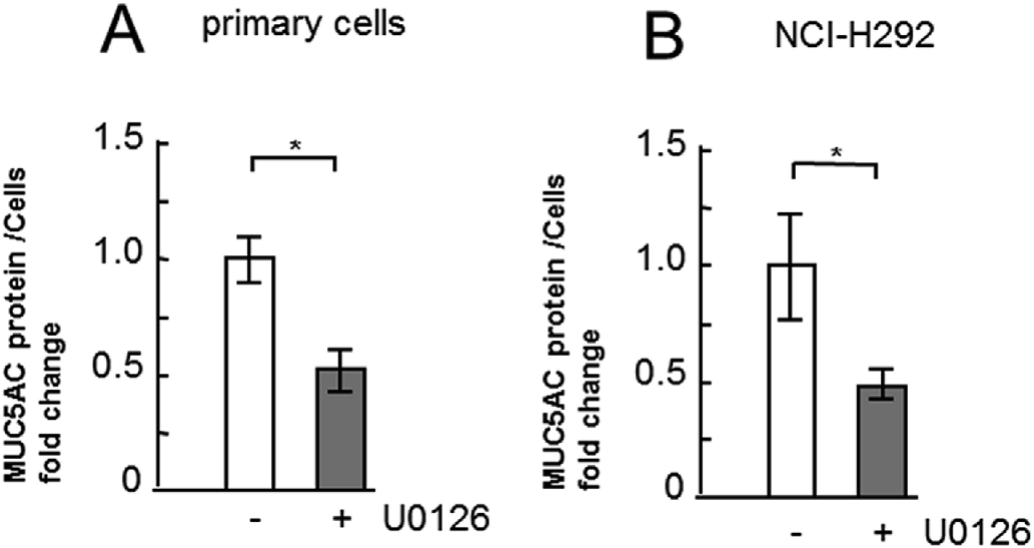
Evaluation of MUC5AC secretion in human primary airway epithelial cells cultured with MEK/ERK inhibitor. Human primary airway epithelial cells were cultured with an MEK/ERK inhibitor (10 μm: U0126: +) or with the same concentration of DMSO (−) for 6 h and sampled. The samples were analyzed using the mucin protein assay to detect the levels of MUC5AC protein. The representative results of 3 independent experiments are shown. Fold changes were based on control level (0 μg/mL of MEK/ERK inhibitor) of secreted MUC5AC in medium (mean ± SD, n = 6, one-way ANOVA).

**Fig. 7:**
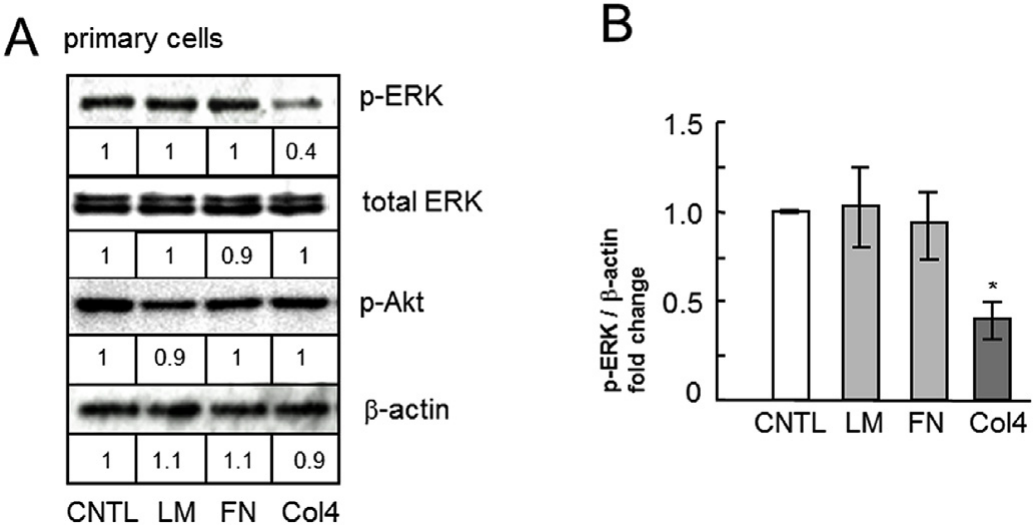
Evaluation of ERK activity in human primary airway epithelial cells cultured with type IV collagen. Human primary airway epithelial cells were cultured with PBS (CNTL), 500 μg/mL of laminin (LM), fibronectin (FN) or type IV collagen (Col4) for 6 h and sampled. The samples were analyzed using Western blot analysis to detect the levels of phosphorylated and activated form of ERK (p-ERK), total ERK, phosphorylated and activated form of Akt (p-Akt) and β-actin. The representative results of 3 independent experiments are shown. The normalized p-ERK, total ERK, β-actin and p-ERK/β-actin intensities were expressed as fold change in comparison to CNTL considered equal to 1. (mean ± SD, n = 3, one-way ANOVA).

To identify the pathway resulting in type IV collagen induced reduction in MUC5AC secretion, the activity of ERK, a representative kinase that induces an increase in MUC5AC secretion, was assessed in human primary airway epithelial cells. The amount of MUC5AC secretion was downregulated by U0126, an inhibitor of the mitogen-activated protein kinase (MEK)/ERK pathway (Fig. 6). This suggested that MUC5AC secretion was dependent on the MEK/ERK pathway in human primary airway epithelial cells. Next, we assessed the activity of ERK and Akt in the primary cells incubated with ECM proteins by measuring ERK activation through Western blot analysis using phospho-specific ERK and phospho-specific Akt antibodies. ERK activity was significantly reduced in cells incubated with type IV collagen compared with that in untreated cells, while there was no considerable change observed in cells incubated with laminin or fibronectin compared with that in untreated cells (Fig. 7). In contrast, Akt activity also showed no considerable change in cells incubated with type IV collagen, laminin, or fibronectin compared with that in untreated cells (Fig. 7).

In the NCI–H292 cell line, Akt downregulated MUC5AC production, and its activity increased markedly in cells cultured with type IV collagen. In the primary cells incubated with type IV collagen, the Akt activity remained unchanged compared with that in untreated cells (Fig. 7).

## 4 Discussion

The airways in the lungs of patients with asthma overreact to various stimuli, which results in airway narrowing and air flow obstruction. Since bronchial contraction and MUC5AC hypersecretion are two important factors that induce airway narrowing, reduction in MUC5AC secretion is important in treating asthma. In this study, ECM proteins regulated MUC5AC secretion in human primary airway epithelial cells which were derived from patients with asthma and in human lung epithelial cell line NCI–H292 (Fig. 1, Fig. 2, Fig. 3, Fig. 4, Fig. 5).

The basement membrane of human primary airway epithelial cells contains accumulated ECM proteins, such as type I, III, and IV collagens, fibronectins, and laminins, which are produced from activated myofibroblasts. In patients with asthma, a considerable thickening of the basement membrane has been observed, and ECM proteins are extensively altered [[26], [27], [28]]. A mice model of asthma showed increased expression of laminin-1 isoform and laminin-1 receptor in the airways [29]. In addition, airway smooth muscle cells in patients with asthma showed an increased mass of ECM proteins and altered ECM profiles [30,31]. Although considerable changes in ECM proteins in the airway epithelia have been observed in patients with asthma, the relationship between these changes and MUC5AC secretion remained unclear. Our results showed that changes in ECM proteins could regulate MUC5AC secretion in human primary airway epithelial cells and suggested that an increase in type IV collagen or decrease in laminin in the asthmatic airway can cause reduction in MUC5AC secretion in vivo.

Further, our results suggest that type IV collagen induces the downregulation of ERK activity and reduces MUC5AC secretion (Fig. 6, Fig. 7). The activation of ERK by ECM proteins is induced by integrin heterodimers expressed in the cell membrane. As a result, the activity of integrin heterodimers might be downregulated by the addition of type IV collagen, and the downregulation of the ERK signaling pathway relates to the reduction in MUC5AC secretion.

Most of our results with primary cells were similar to those with NCI–H292 cells, with the exception of Akt activity, which remained unchanged with the addition of type IV collagen to primary cells and was activated in NCI–H292 cells (Fig. 7). However, the direction showing the effect of type IV collagen on cells was completely different. In the experiments with NCI–H292 cells cultured on type IV collagen-coated plates, the effects of type IV collagen come from the cell adhesion surface. In contrast, in the experiments with three-dimensional cultured primary cells, the effects of applied type IV collagen come from the upper phase of the overlapped cell layer, which mimics the surface of the human airway. In the future, to treat patients with asthma, we plan to administer nebulization of type IV collagen. Thus, the determination of the effects of type IV collagen on the airway surface is essential.

**Fig. 8:**
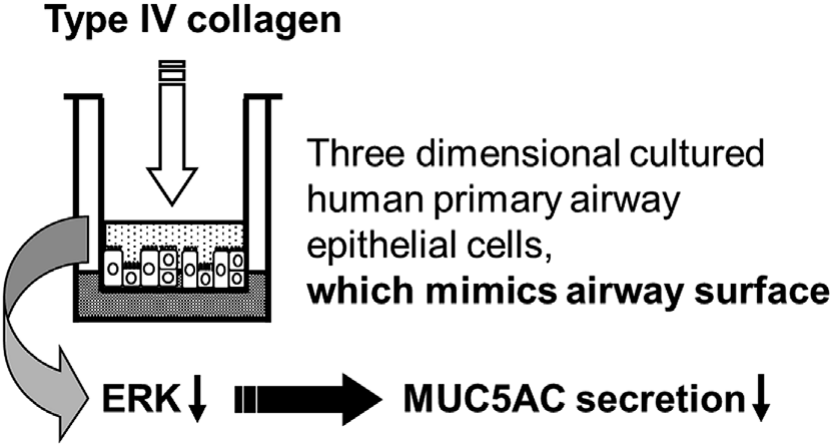
Graphical abstract. The addition of type IV collagen to the apical surface of human primary airway epithelial cells downregulate MUC5AC secretion and type IV collagen has cumulative effect on MUC5AC secretion which might work via the ERK signaling pathway.

Our results suggest that the nebulization of type IV collagen to the apical surface of bronchial tubes could be sufficient to reduce MUC5AC hypersecretion in vivo (Fig. 8). Further, the repeated addition of low levels of type IV collagen could show cumulative effects and could effectively reduce MUC5AC levels (Fig. 5). An analysis of the effects of repeated nebulization of low levels of type IV collagen in a mouse model of asthma will be required. Therefore, our results could relate to an effective treatment method to reduce airway mucus secretion through repeated nebulization of low levels of type IV collagen in patients with asthma.

## Funding information

Akita Prefectural University President's Research Project Fund (A52120123), MEXT/JSPS KAKENHI Grant Number JP19K05882.

## Declaration of competing interest

The authors declare no conflicts of interest involving this article.

## References

[1] Aikawa T., Shimura S., Sasaki H., Ebina M., Takishima T. (1992). Marked goblet cell hyperplasia with mucus accumulation in the airways of patients who died of severe acute asthma attack. Chest.

[2] Vestbo J. (2002).

[3] Rogers D.F. (2003). The airway goblet cell. Int. J. Biochem. Cell Biol..

[4] Voynow J.A., Gendler S.J., Rose M.C. (2006). Regulation of mucin genes in chronic inflammatory airway diseases. Am. J. Respir. Cell Mol. Biol..

[5] Rose M.C., Voynow J.A. (2006). Respiratory tract mucin genes and mucin glycoproteins in health and disease. Physiol. Rev..

[6] Fahy J.V. (2002). Goblet cell and mucin gene abnormalities in asthma. Chest.

[7] Rogers D.F. (2004). Airway mucus hypersecretion in asthma: an undervalued pathology?. Curr. Opin. Pharmacol..

[8] Wang K., Wen F.Q., Xu D. (2008). Mucus hypersecretion in the airway. Chin. Med. J..

[9] Perez-Vilar J., Sheehan J.K., Randell S.H. (2003). Making more MUCS. Am. J. Respir. Cell Mol. Biol..

[10] Iwashita J., Ose K., Ito H., Murata J., Abe T. (2011). Inhibition of E-cadherin dependent cell-cell contact promotes MUC5AC mucin production through the activation of epidermal growth factor receptors. Biosci. Biotechnol. Biochem..

[11] Iwashita J., Ito Y., Yokoo M., Takahashi S., Murata J. (2014). Akt induces down regulation of MUC5AC production in NCI-H292 human airway epithelial cells cultured on extracellular matrix. Biosci. Biotechnol. Biochem..

[12] Iwashita J., Iguchi N., Takashima A., Watanabe D., Sano K., Ishikuro M., Hata K., Murata J. (2017). Citrus jabara extracts suppress MUC5AC mucin production in human lung epithelial cells. Adv. Biol. Chem..

[13] Chen Y., Thai P., Zhao Y.H., Ho Y.S., DeSouza M.M., Wu R. (2003). Stimulation of airway mucin gene expression by interleukin (IL)-17 through IL-6 paracrine/autocrine loop. J. Biol. Chem..

[14] Song K.S., Lee W.J., Chung K.C., Koo J.S., Yang E.J., Choi J.Y., Yoon J.H. (2003). Interleukin-1 beta and tumor necrosis factor-alpha induce MUC5AC overexpression through a mechanism involving ERK/p38 mitogen-activated protein kinases-MSK1-CREB activation in human airway epithelial cells. J. Biol. Chem..

[15] Takeyama K., Dabbagh K., Jeong Shim J., Dao-Pick T., Ueki I.F., Nadel J.A. (2000). Oxidative stress causes mucin synthesis via transactivation of epidermal growth factor receptor: role of neutrophils. J. Immunol..

[16] Basbaum C., Li D., Gensch E., Gallup M., Lemjabbar H. (2002). Mechanisms by which gram-positive bacteria and tobacco smoke stimulate mucin induction through the epidermal growth factor receptor (EGFR). Novartis Found. Symp..

[17] Takeyama K., Dabbagh K., Lee H.M., Agusti C., Lausier J.A., Ueki I.F., Grattan K.M., Nadel J.A. (1999). Epidermal growth factor system regulates mucin production in airways. Proc. Natl. Acad. Sci. U.S.A..

[18] Bozulic L., Hemmings B.A. (2009). PIKKing on PKB: regulation of PKB activity by phosphorylation. Curr. Opin. Cell Biol..

[19] Hemmings B.A., Restuccia D.F. (2015). The PI3K-PKB/Akt pathway. Cold Spring Harb. Perspect. Biol..

[20] Ingber D.E., Dike L., Hansen L., Karp S., Liley H., Maniotis A., McNamee H., Mooney D., Plopper G., Sims J. (1994). Cellular tensegrity: exploring how mechanical changes in the cytoskeleton regulate cell growth, migration, and tissue pattern during morphogenesis. Int. Rev. Cytol..

[21] Coppock H.A., Gilham D.E., Howell A., Clarke R.B. (2007). Cyclin-dependent kinase inhibitors and basement membrane interact to regulate breast epithelial cell differentiation and acinar morphogenesis. Cell Prolif.

[22] Wilhelmsen K., Litjens S.H., Kuikman I., Margadant C., van Rheenen J., Sonnenberg A. (2007). Serine phosphorylation of the integrin beta4 subunit is necessary for epidermal growth factor receptor induced hemidesmosome disruption. Mol. Biol. Cell.

[23] Segal N., Andriani F., Pfeiffer L., Kamath P., Lin N., Satyamurthy K., Egles C., Garlick J.A. (2008). The basement membrane microenvironment directs the normalization and survival of bioengineered human skin equivalents, Matrix biology. J. Int. Soc. Matrix Biol..

[24] Yang B.G., Tanaka T., Jang M.H., Bai Z., Hayasaka H., Miyasaka M. (2007). Binding of lymphoid chemokines to collagen IV that accumulates in the basal lamina of high endothelial venules: its implications in lymphocyte trafficking. J. Immunol..

[25] Iwashita J., Yamamoto T., Sasaki Y., Abe T. (2010). MUC5AC production is downregulated in NCI-H292 lung cancer cells cultured on type-IV collagen. Mol. Cell. Biochem..

[26] Roche W.R., Beasley R., Williams J.H., Holgate S.T. (1989). Subepithelial fibrosis in the bronchi of asthmatics. Lancet.

[27] Altraja A., Laitinen A., Virtanen I., Kampe M., Simonsson B.G., Karlsson S.E., Hakansson L., Venge P., Sillastu H., Laitinen L.A. (1996). Expression of laminins in the airways in various types of asthmatic patients: a morphometric study. Am. J. Respir. Cell Mol. Biol..

[28] Chakir J., Laviolette M., Boutet M., Laliberte R., Dube J., Boulet L.P. (1996). Lower airways remodeling in nonasthmatic subjects with allergic rhinitis. Lab. Investig..

[29] Christie P.E., Jonas M., Tsai C.H., Chi E.Y., Henderson W.R. (2004). Increase in laminin expression in allergic airway remodelling and decrease by dexamethasone. Eur. Respir. J..

[30] Chung K.F. (2000). Airway smooth muscle cells: contributing to and regulating airway mucosal inflammation?. Eur. Respir. J..

[31] Johnson P.R. (2001). Role of human airway smooth muscle in altered extracellular matrix production in asthma. Clin. Exp. Pharmacol. Physiol..

